# Supplementary material for: Energy and environmental impacts of air-to-air heat pumps in a mid-latitude city
Source: Nat Commun. 2024 Jun 28;15:5474. doi: 10.1038/s41467-024-49836-3 (PMC11213923; doi:10.1038/s41467-024-49836-3)
Supplement: Supplementary file 1 — Supplementary Information [file 41467_2024_49836_MOESM1_ESM.pdf]

Supplementary Information for "Energy and environmental impacts of air-to-air heat pumps in a mid-latitude city"

David Meyer[1,*] (ORCID: 0000-0002-7071-7547)

Robert Schoetter[2] (ORCID: 0000-0002-2284-4592)

Maarten van Reeuwijk[1] (ORCID: 0000-0003-4840-5050)

[1]Department of Civil and Environmental Engineering, Imperial College London, London, UK

[2]CNRM, Université de Toulouse, Météo-France, CNRS, Toulouse, France

*Correspondence to David Meyer (email: d.meyer@imperial.ac.uk)

Contents:

- Supplementary Figures 1-9

Supplementary Figures

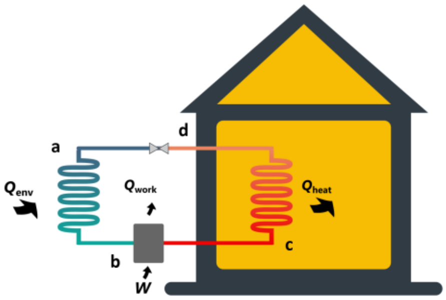

Supplementary Fig. 1 | Schematic of the vapour–compression refrigeration cycle used by an electrically-driven AAHP as implemented in this study. (**a**) The refrigerant absorbs thermal energy (Q_{env}) from the environment through the external coil. (**b**) This low-pressure, low-temperature refrigerant then moves to the compressor where work (W) is done to increase its pressure and temperature. (**c**) The now high-pressure, high-temperature refrigerant moves to the indoor coil, where it releases its thermal energy (Q_{heat}) to heat the indoor air inside the building through the internal coil. (**d**) Following this heat release, the refrigerant, still under high pressure but now at a lower temperature, passes through the expansion valve, where its pressure decreases, readying it to absorb heat from the environment once more, thereby continuing the cycle.

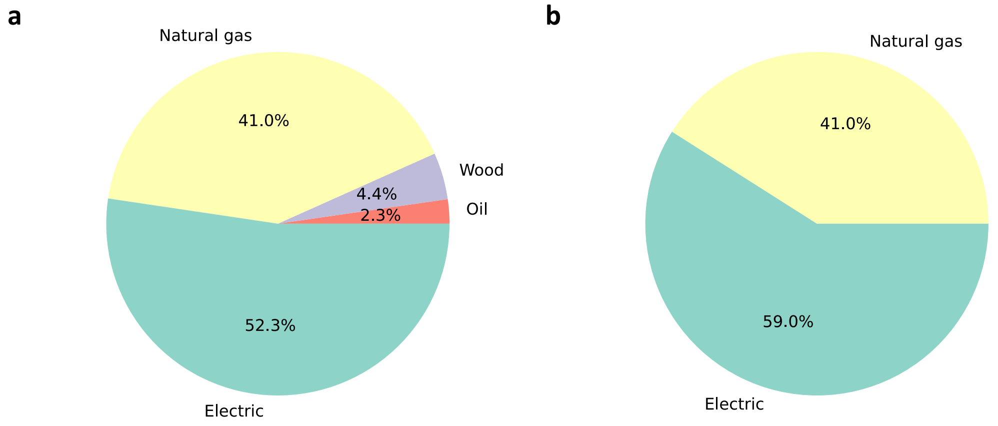

Supplementary Fig. 2 | Percentage of heating fuel types in the domain of investigation. These are shown for (**a**) the entire domain and (**b**) for the dense urban centre of Toulouse (Fig. 6b and Fig. 6c, respectively).

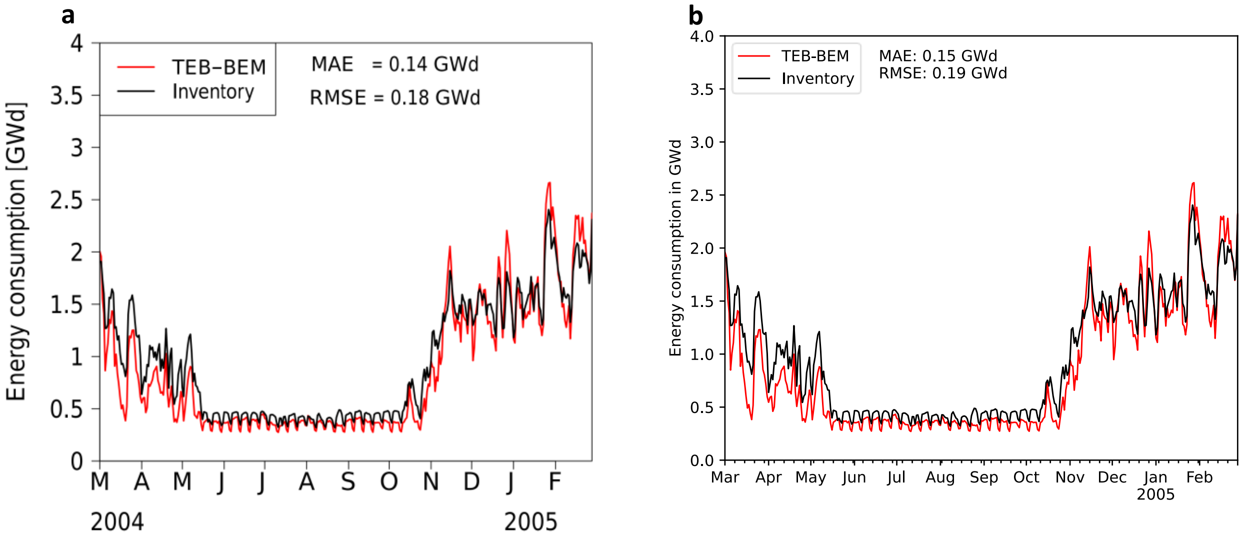

Supplementary Fig. 3 | Evaluation of offline SURFEX-TEB model's baseline scenario. Time series of daily building energy consumption consisting of heating, cooling, domestic warm water, cooking, lighting, and electrical appliances within the offline simulation domain from 1 March 2004 to 28 February 2005 with (**a**) the simulation results presented in Fig. 6d ref.[1] (licensed under Creative Commons Attribution 3.0) and (**b**) our results closely aligning with their findings. Minor discrepancies of 0.01 GWd in Mean Absolute Error (MAE) and Root Mean Square Error (RMSE) are attributed to updated map inputs used in offline simulations. As noted in ref.[1], the baseline's building energy consumption is well represented. The building energy consumption during the warm period averages at about 0.4 GWd, revealing a weekly pattern due to decreased occupancy in offices and commercial spaces on weekends. Energy consumption during this period shows minimal temperature dependence, reflecting the scarce presence of air conditioners in Toulouse in the summer of 2004.

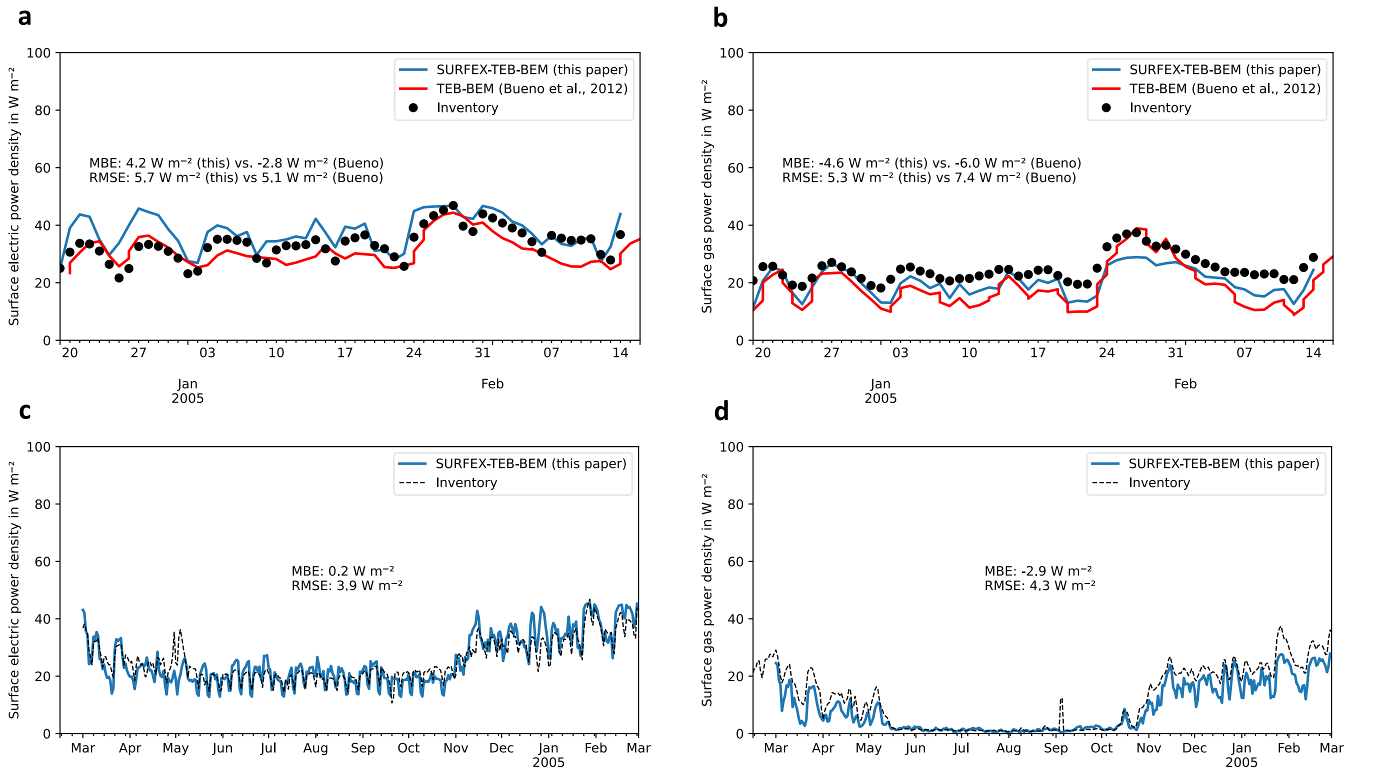

Supplementary Fig. 4 | Evaluation of winter (December, January, February) daily averaged surface power density in Toulouse's urban centre. (**a**) electrical and (**b**) natural gas power densities, normalized by urban area, are compared with results from ref.[2] and inventory[3]. The mean bias error (MBE) and root mean square error (RMSE) are computed against inventory. These are lower or on par with those presented in ref.[2]. Data for plotting TEB-BEM was extracted from ref.[2] Fig. 9. (**c**) electric and (**d**) natural gas power densities, normalized by urban area, are compared with observed values from the dense core of Toulouse for the entire year.

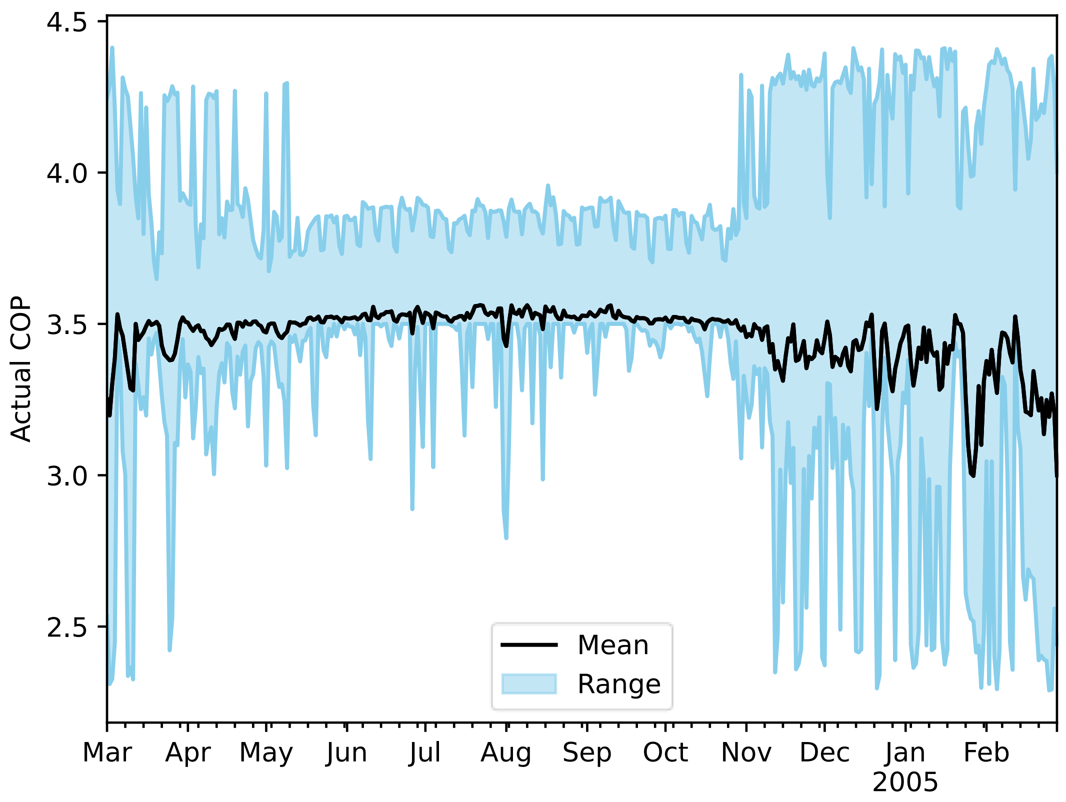

Supplementary Fig. 5 | Time series of simulated COP for the entire Toulouse agglomeration. The RC3.5 scenario's simulated COP is shown for mean (black line) and range (blue shaded area).

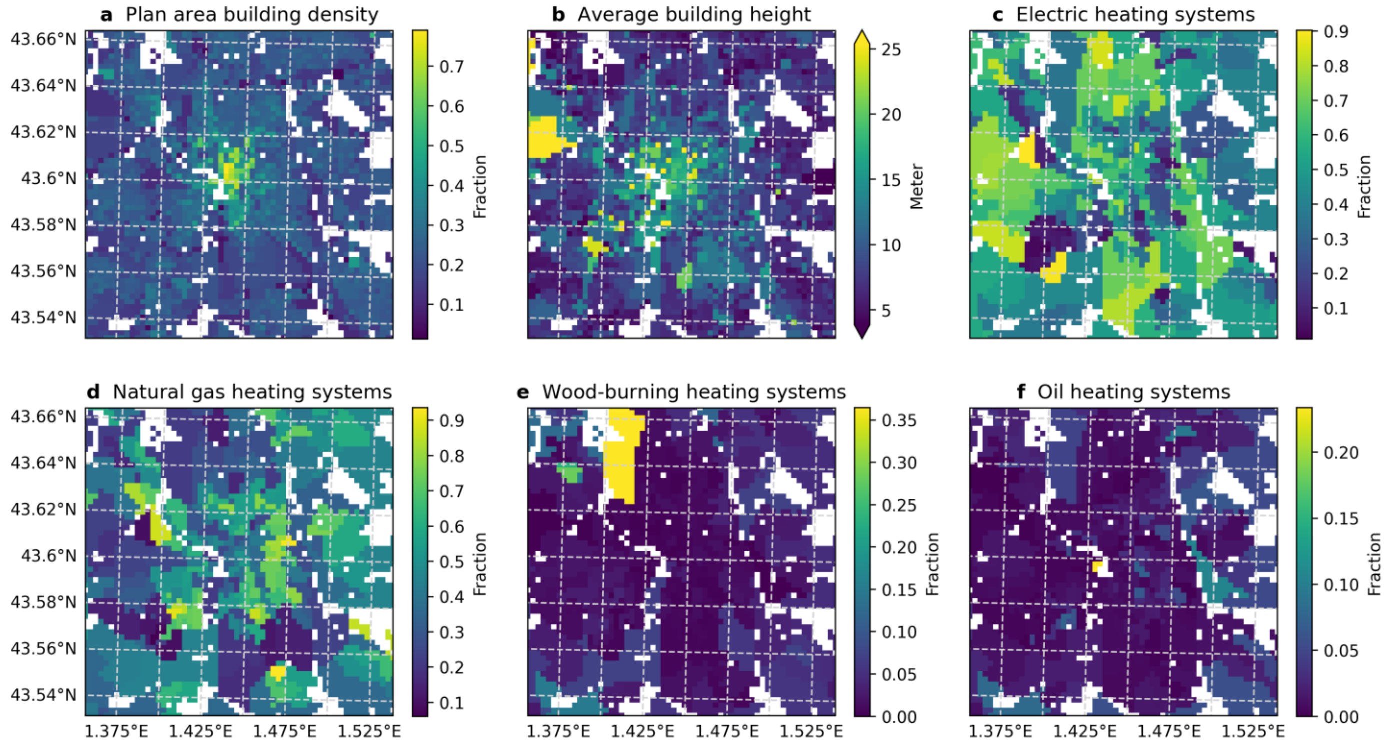

Supplementary Fig. 6 | Input parameters on urban morphology and baseline heating fuel used in TEB. Here shown for (**a**) plan area building density, (**b**) average building height, and heating fuel types such as (**c**) electric, (**d**) natural gas, (**e**) wood, and (**f**) oil.

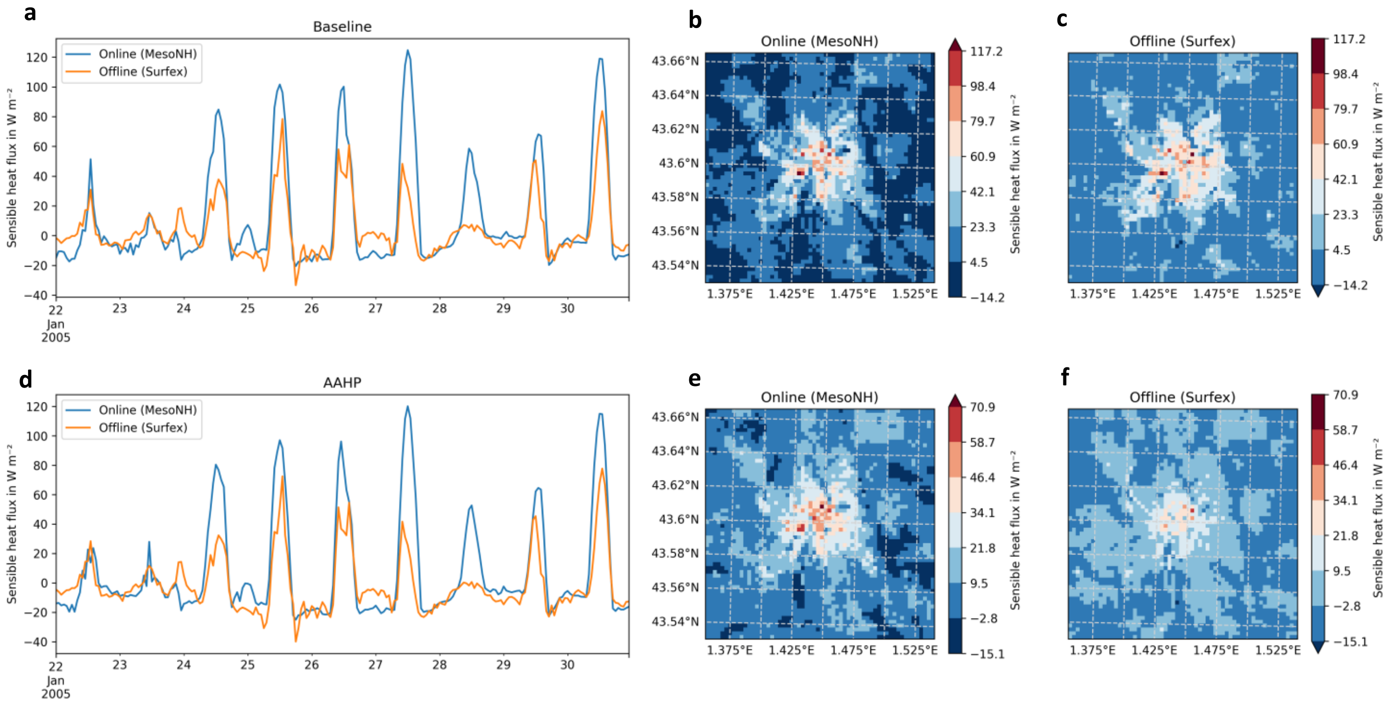

Supplementary Fig. 7 | Comparison of offline and online sensible heat flux during the cold spell period. These are shown for (**a-c**) baseline and (**d-f**) AAHP RC3.5 scenario.

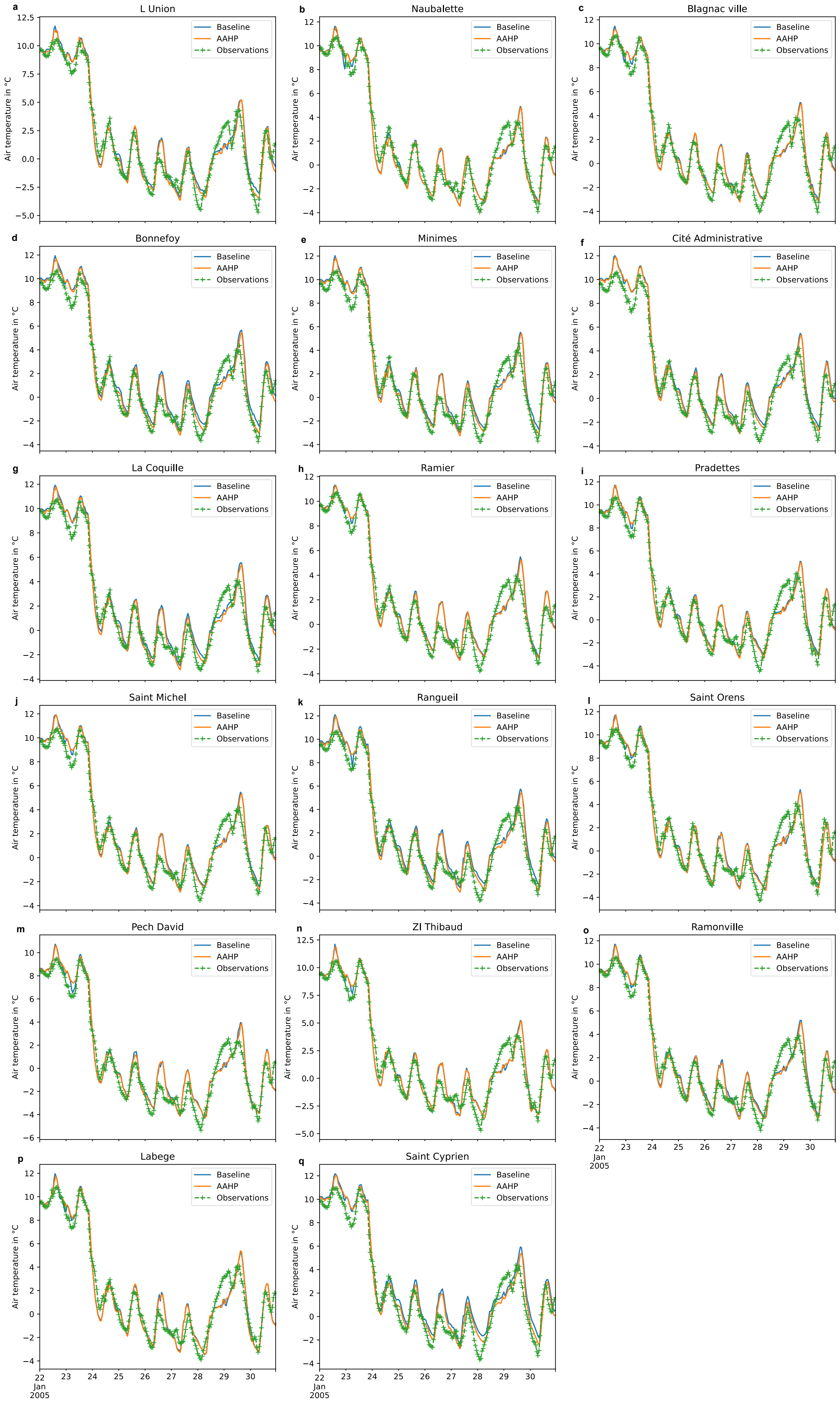

Supplementary Fig. 8 | Comparison of simulated and observed near-surface air temperature for the 17 meteorological stations (Fig. 6b) in Toulouse. Online simulation results for baseline and AAHP RC3.5 are shown alongside observations for (**a**) L'Union, (**b**) Naubalette, (**c**) Blagnac-ville, (**d**) Bonnefoy, (**e**) Minimes, (**f**) Cité Administrative, (**g**) La Coquille, (**h**) Ramier, (**i**) Pradettes, (**j**) Saint Michel, (**k**) Rangueil, (**l**) Saint Orens, (**m**) Pech David, (**n**) ZI Thibaud, (**o**) Ramonville, (**p**) Labege, (**q**) Saint Cyprien.

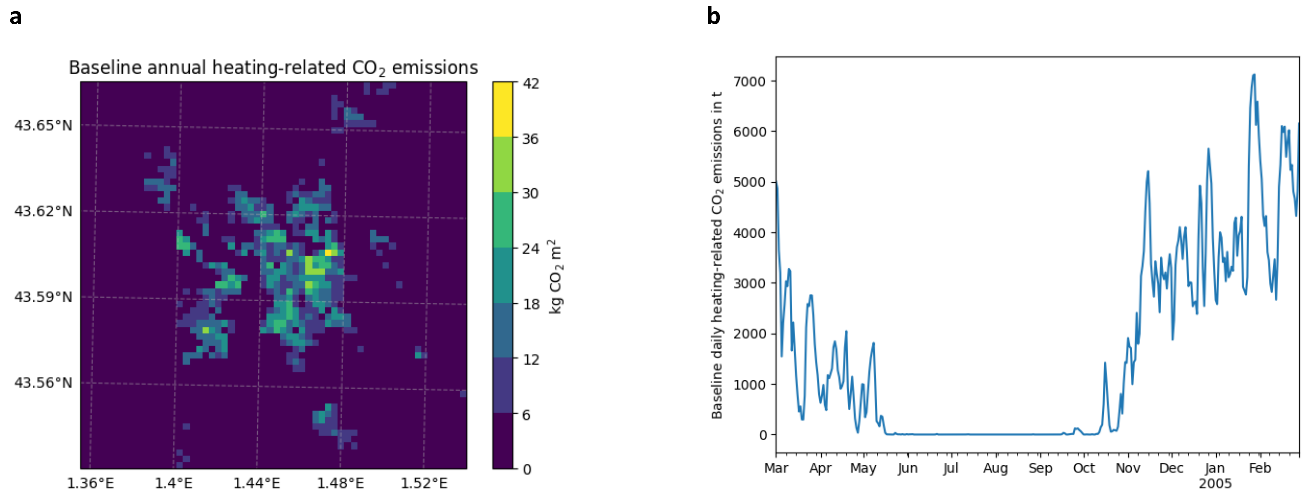

Supplementary Fig. 9 | Baseline heating-related CO_2 emissions. (**a**) Spatial distribution of yearly (March 2004 to February 2005) local CO_2 emissions due to gas heating energy consumption and (**b**) time series of daily CO_2 emissions for the domain displayed in (a). Energy is converted to CO_2 using gas emission factor of 7.5 x 10^{-8} kg CO_2 J^{-1} from ref.[4], Table 1.

Supplementary References

1. Schoetter, R., Masson, V., Bourgeois, A., Pellegrino, M. & Lévy, J.-P. Parametrisation of the variety of human behaviour related to building energy consumption in the Town Energy Balance (SURFEX-TEB v. 8.2). *Geosci. Model Dev.* **10**, 2801–2831 (2017).
2. Bueno, B., Pigeon, G., Norford, L. K., Zibouche, K. & Marchadier, C. Development and evaluation of a building energy model integrated in the TEB scheme. *Geosci. Model Dev.* **5**, 433–448 (2012).
3. Pigeon, G., Legain, D., Durand, P. & Masson, V. Anthropogenic heat release in an old European agglomeration (Toulouse, France). *Int. J. Climatol.* **27**, 1969–1981 (2007).
4. Goret, M., Masson, V., Schoetter, R. & Moine, M.-P. Inclusion of CO2 flux modelling in an urban canopy layer model and an evaluation over an old European city centre. *Atmospheric Environment: X* **3**, 100042 (2019).
